# Novel insights into the role of acetyl-CoA producing enzymes in epigenetic regulation

**DOI:** 10.3389/fendo.2023.1272646

**Published:** 2023-09-29

**Authors:** Marta Russo, Francesco Pileri, Serena Ghisletti

**Affiliations:** Department of Experimental Oncology, European Institute of Oncology (IEO) IRCCS, Milan, Italy

**Keywords:** inflammation, macrophages, acetyl-CoA, mitochondrial enzymes, nitric oxide, transcription, chromatin

## Abstract

Inflammation-dependent changes in gene expression programs in innate immune cells, such as macrophages, involve extensive reprogramming of metabolism. This reprogramming is essential for the production of metabolites required for chromatin modifications, such as acetyl-CoA, and regulate their usage and availability impacting the macrophage epigenome. One of the most transcriptionally induced proinflammatory mediator is nitric oxide (NO), which has been shown to inhibit key metabolic enzymes involved in the production of these metabolites. Recent evidence indicates that NO inhibits mitochondrial enzymes such as pyruvate dehydrogenase (PDH) in macrophages induced by inflammatory stimulus. PDH is involved in the production of acetyl-CoA, which is essential for chromatin modifications in the nucleus, such as histone acetylation. In addition, acetyl-CoA levels in inflamed macrophages are regulated by ATP citrate lyase (ACLY) and citrate transporter SLC25A1. Interestingly, acetyl-CoA producing enzymes, such as PDH and ACLY, have also been reported to be present in the nucleus and to support the local generation of cofactors such as acetyl-CoA. Here, we will discuss the mechanisms involved in the regulation of acetyl-CoA production by metabolic enzymes, their inhibition by prolonged exposure to inflammation stimuli, their involvement in dynamic inflammatory expression changes and how these emerging findings could have significant implications for the design of novel therapeutic approaches.

## Introduction

During the inflammatory response, specific cells of the innate immune system, such as macrophages, serve as an internal defense mechanism against infection and tissue damage.

By recognizing pathogen-associated molecular patterns, macrophages undergo significant changes in gene expression programs and activate an extensive reprogramming of their metabolism (1–3). Recent research has shed light on the intricate relationship between transcription factors and chromatin modifications that contribute to the activation of proinflammatory mediators in response to inflammatory stimuli. It is well established that dynamic change of macrophage activation is directed by the activation of specific transcription factors (TFs). Macrophage lineage-determining TFs such as PU.1 and IRF8 and signal-regulated TFs (such as NFkB and STATs family members) bind to genomic regulatory regions to enable different gene expression programs (4–11). In this way, when exposed to pro-inflammatory stimuli such as lipopolysaccharide (LPS) and interferon gamma (IFNg), macrophages undergo classical activation, which results in the expression of proinflammatory cytokines, chemokines, reactive oxygen species (ROS), and microbicidal molecules such as nitric oxide (NO). Moreover, inflammatory conditions lead to the transcriptional upregulation of genes responsible for metabolic pathways. Recently, it has been established that metabolic reprogramming plays a significant role in macrophage pro-inflammatory response, including the upregulation of glycolysis, modifications to the tricarboxylic acid (TCA) cycle, and the impairment of mitochondrial respiration (12–14). Briefly, the remodeling of TCA cycle in the activation of classical macrophages is primarily driven by the tight regulation by the inflammatory stimulus of aconitate decarboxylase 1 (ACOD1) (15), generating the metabolite itaconate, which exerts various immunoregulatory effects (16–19). In turn, accumulation of itaconate inhibits succinate dehydrogenase (SDH), resulting also in succinate accumulation (13, 20). The discussion on how changes of metabolites levels impact on epigenetic regulation in inflammatory states are outside of our main focus and have been extensively covered in recent review articles (3, 21–26).

In this Mini Review, we aim to discuss emerging concepts related to metabolic reprogramming and transcriptional activation in macrophages induced by inflammatory stimuli. Specifically, we will focus on how nitric oxide, produced by macrophages upon prolonged exposure to LPS, affects metabolic remodeling and consequently regulates the transcription of inflammatory genes. Recent studies have revealed the mechanism by which NO blocks the activity of mitochondrial TCA cycle enzymes, including pyruvate dehydrogenase (PDH), which impacts acetyl-CoA production (27–29). acetyl-CoA content, in inflamed macrophages, is also dependent on citrate accumulated in mitochondria, which is transported to the cytosol by SLC25A1, a citrate-malate transporter, and then cleaved into oxaloacetate and acetyl-CoA by ATP citrate lyase (ACLY) in the cytosol. Thus, we will also explore the roles of SLC25A1 and ACLY in regulating acetyl-CoA levels in macrophages exposed to LPS, which can impact histone acetylation and inflammatory gene expression (30–32). Furthermore, we will discuss how enzymes that produce acetyl-CoA, such as PDH and ACLY, can be recruited to the nucleus to generate acetyl-CoA locally, which in turn can impact histone acetylation and transcriptional activation of proinflammatory mediators (33, 34). Finally, we will discuss how these emerging findings have implications for the development of new therapeutic strategies to treat inflammatory diseases.

## Nitric oxide is a central player in inflammation-induced metabolism remodeling

Central to the inflammatory response in macrophages is the transcriptional induction of inducible nitric oxide synthase (iNOS), which produces significant amounts of nitric oxide (NO) and reactive nitrogen species (RNS). One of the essential steps for iNOS transcriptional activation is the binding of transcription factors induced by inflammatory stimuli on iNOS promoter region, such as NF-kB, AP1, IRFs and STATs family members (35). Inflammatory macrophages convert arginine into NO through iNOS activity, being this crucial for host defense and pathogen killing (36). Recent studies suggest that the production of nitric oxide plays a critical role in the metabolic reprogramming of macrophages during activation.

**Figure 1:**
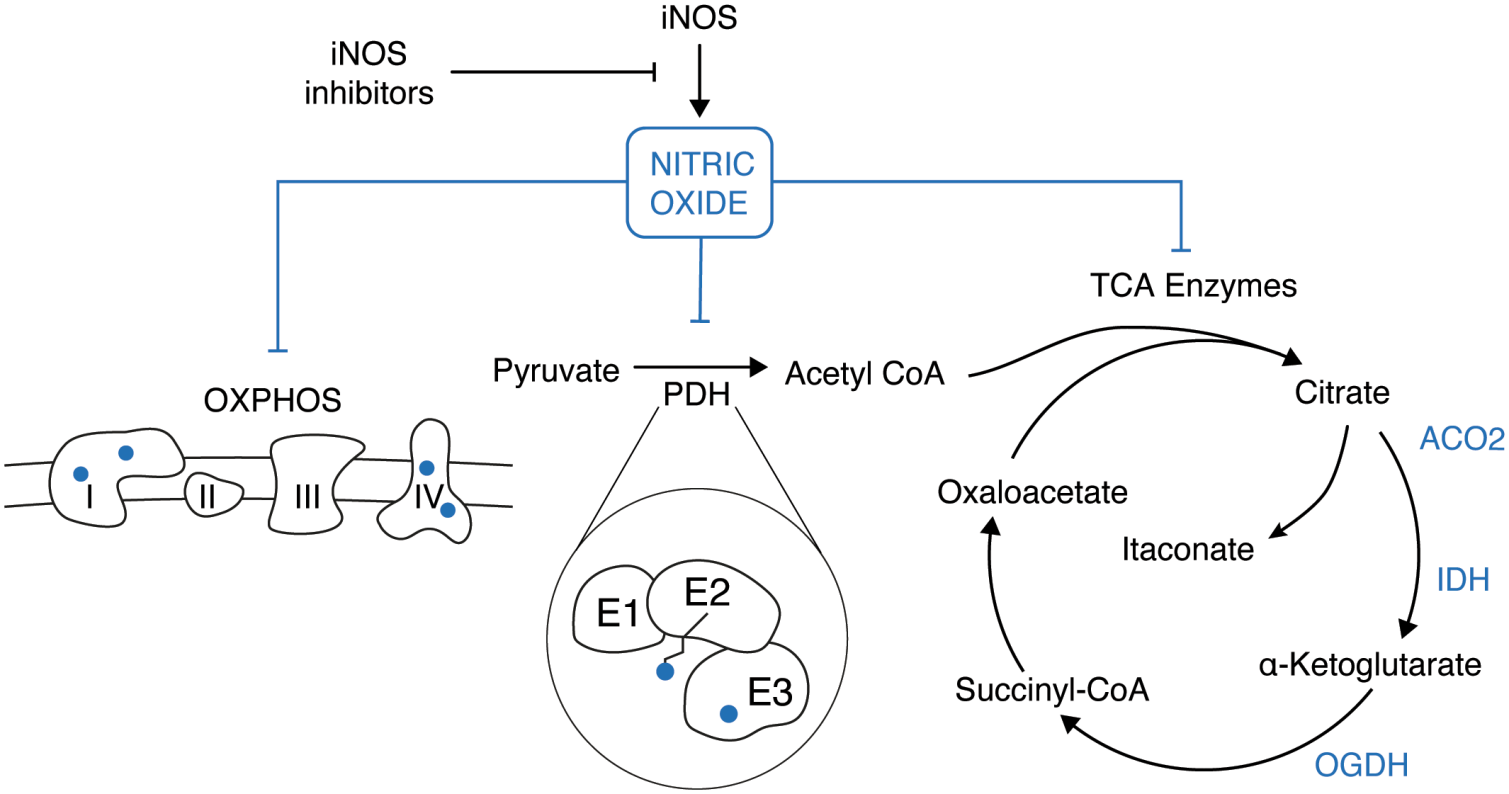
In LPS-activated macrophages the induction of the iNOS gene results in a burst of nitric oxide (NO) production. NO has a pleiotropic effect inside the cell which results in the inhibition of the electron transport chain (OXPHOS), the E2 and E3 subunit of the pyruvate dehydrogenase (PDH), and few enzymes of the TCA cycle (ACO2, IDH, OGDH).

First of all, the generation of NO plays a key role in the pro-inflammatory switch of macrophages by inhibiting mitochondrial respiration. This is achieved through nitrosation of NADH dehydrogenase (Complex I) and reversible inhibition of cytochrome c oxidase (Complex IV) (37–39), with recent evidence indicating that NO primarily decreases the activity of complexes I and II, with minor effects on complexes III and IV (14). Thus, NO and NO-derived reactive nitrogen species can inactivate all iron-sulfur-containing complexes of the mitochondrial transport chain, thereby inhibiting electron transport and ATP production (14) (**Figure 1**).

Secondly, in addition to its effects on mitochondrial respiration, NO also modulates metabolic remodeling in inflammatory macrophages by regulating the TCA cycle. Specifically, NO has been shown to inhibit pyruvate dehydrogenase (PDH) (27, 28) (**Figure 1**), which is part of the mitochondrial a-ketoacid dehydrogenase family of multi-subunit enzyme complexes that also includes oxoglutarate dehydrogenase (OGDH) and branched-chain ketoacid dehydrogenase complex (BCKDC). These complexes have a similar catalytic mechanism involving coupled reactions with three subunits: E1, E2, and E3. The E1 subunit decarboxylates an a-ketoacid and transfers the corresponding acyl group to a thiamine pyrophosphate cofactor. The E2 subunit (dihydrolipoamide acyltransferase) then transfers the acyl group to the thiol of CoA, producing acyl-CoA, and the E3 subunit (dihydrolipoamide dehydrogenase) reoxidizes the lipoic arm, coupled to NADH production (40).

Mechanisms targeting different subunits of PDH have been recently proposed to regulate its activity (**Figure 1**). One such mechanism involves the generation of reactive nitrogen species (RNS), which induce covalent S-modifications on the lipoic arm of the PDH E2 subunit (29). This results in the formation of adducts that hinder catalytic activity, and this mechanism appears to be highly specific and effective due to the targeted delivery of RNS modifications on the lipoic arm via CoA (27, 29). As alternative and complementary mechanism, also the E3 subunit can be directly nitrosylated by NO in LPS activated macrophages (28). It is important to note that the same mechanisms here described for PDH can be applied also for other enzymes belonging to the same family, such as OGDH and BCKDC (**Figure 1**). Moreover, NO has been demonstrated to inhibit aconitase (ACO2) and other enzymes of the TCA cycle, such as isocitrate dehydrogenase (IDH) (39, 41, 42) (**Figure 1**).

In this manner, NO plays a unique and fundamental role in regulating the balance of the key metabolites for macrophage function such as acetyl-CoA, itaconate, succinate and citrate. Thus, NO takes center stage not only as an orchestrator of changes in macrophage mitochondrial metabolism during prolonged stimulation, but also a potential regulator of downstream epigenomic changes.

## SLC25A1 and ACLY regulate inflammatory gene expression

**Figure 2:**
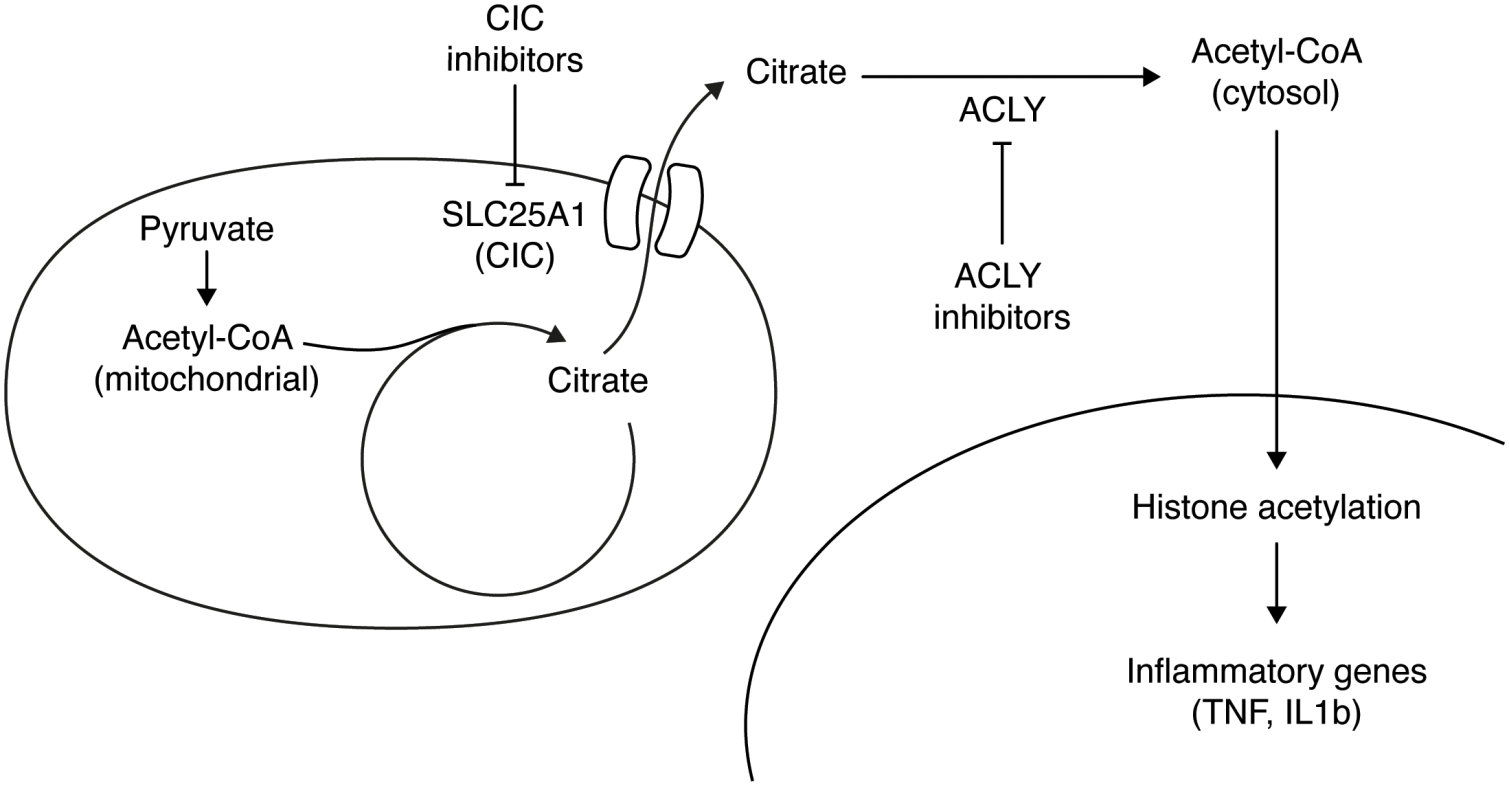
Citrate accumulates in the mitochondria of LPS-activated macrophages and it is exported to the cytosol through its carrier (SLC25A1). In the cytosol ATP Citrate Lyase (ACLY) converts citrate to acetyl-CoA, which is used in the nucleus to acetylate histones and thus activate target inflammatory genes. Inhibiting either ACLY activity or the citrate transporter results in a reduction of inflammatory genes activation.

During the early stages of an inflammatory response, there is an upregulation of glycolysis, leading to an increased production of pyruvate. This pyruvate is then utilized by PDH to enter the TCA cycle (30). Simultaneously, the inflammatory stimulus causes a transcriptional downregulation of isocitrate dehydrogenase (IDH) enzyme, resulting in the accumulation of mitochondrial citrate (12, 13). To maintain appropriate levels of acetyl-CoA, the accumulated mitochondrial citrate is exported to the cytosol, where it undergoes cleavage by ACLY, in an ATP-consuming reaction that generates oxaloacetate and acetyl-CoA (**Figure 2**). Notably, macrophages lack the expression of acyl-CoA synthase short-chain family member 2 (ACSS2), which is responsible for converting acetate to acetyl-CoA, representing the major route to cytosolic acetyl-CoA that does not involve citrate. Inflammatory macrophages show activation of ACLY, as evidenced by an increase in its phosphorylation, despite no changes in gene expression or protein levels (30, 31, 43). Acetyl-CoA generated by ACLY can be incorporated into histones, thereby promoting chromosome accessibility and regulating macrophage activation induced by LPS and IL4 (30, 44) (**Figure 2**).

An additional layer of regulation of acetyl-CoA levels in inflammatory macrophages involves citrate transport. In this regard, the mitochondrial citrate carrier (CIC), also known as SLC25A1, plays crucial role in exporting citrate/isocitrate from the mitochondria in exchange for the entry of cytosolic malate (23) (**Figure 2**). The induction of CIC and the export of citrate have been shown to regulate inflammatory mediators in macrophages (45).

Recent discoveries have highlighted the significance of exported citrate and cytosolic acetyl-CoA generation as signaling metabolites that govern metabolite reprogramming to support inflammatory responses (32). In activated macrophages, inhibiting CIC promotes metabolic flux in the TCA cycle, leading to a reduction in mitochondrial citrate and succinate accumulation, which in turn suppresses inflammatory responses at the metabolic level (32). Blocking CIC or treating with an ACLY inhibitor impaired the LPS-induced production of proinflammatory mediators such as interleukin1b (IL1b), iNOS, tumor necrosis factor (TNF) and prostaglandin E2 (PGE2) (30, 31, 46) (**Figure 2**).

## Acetyl-CoA producing enzymes act at a nuclear level

Acetyl-CoA, a molecule that faces difficulty in crossing cellular membranes, has two possible ways to enter the nucleus: either through nuclear pores or by being produced directly within the nucleus. Similarly, citrate, which has the ability to diffuse across nuclear pores, can be utilized within the nucleus to generate acetyl-CoA specifically for histone acetylation.

Interestingly, the cytosolic enzyme ACLY is present in the nucleus and plays a role in enhancing histone acetylation, thereby promoting the transcriptional activation of specific genes (47). Although the regulation of histone acetylation levels primarily relies on histone acetyltransferases and deacetylases, it has been recognized that acetyl-CoA derived from exported citrate also contributes to the process of histone acetylation. Consequently, the nucleus is now acknowledged as an active metabolic compartment where the generation of acetyl-CoA takes place.

Besides ACLY, also the other acetyl-CoA producing enzyme PDH has been extensively demonstrated to be present and functional in the nucleus. Despite its primary localization in the mitochondria, PDH has been reported to undergo translocation from the mitochondria to the nucleus in response to specific stimuli, thereby playing a role in histone acetylation regulation (34, 48–52).

The challenge lies in understanding how this large macromolecular enzyme complex, which can reach sizes up to 10 MDa, is able to traverse from the mitochondria to the nucleus, considering its inability to pass through nuclear pores. As it lacks a nuclear localization signal (NLS), the mechanism behind PDH’s translocation remains unclear. Recent studies have proposed a non-canonical nuclear import pathway that does not involve the nuclear pore complex (34). In this proposed mechanism, PDH translocation is facilitated by tethering the mitochondria to the nucleus through the involvement of Mitofusin 2 (MFN2), a protein crucial for regulating mitochondrial fusion (34, 53).

Interestingly, a recent biochemical study utilizing mass spectrometry has provided intriguing findings regarding the flexibility of PDH’s structure. It was discovered that under physiological ionic strength, PDH exhibits pliability, allowing it to change its size and dissociate into sub-megadalton individual components (33). These findings suggest that PDH is a dynamic complex capable of dissociating into smaller components, which may facilitate its translocation into the nucleus. In addition, it has been recently reported that also aconitase 2 and 2-oxoglutarate dehydrogenase are present and act at a nuclear level (54). By proximity labeling mass spectrometry, these enzymes have been shown to be spatially close to nuclear proteins (54).

Further exploration into the mechanisms underlying the translocation of metabolic enzymes into the nucleus, as well as their involvement in epigenetic regulation in activated macrophages, holds the potential to unveil novel insights into the intricate interplay between metabolism and gene expression.

## Discussion

The existence of such an intricate interplay between cellular metabolism and macrophage transcriptional response has led to the exploration of metabolic reprogramming as a novel therapeutic strategy for controlling macrophage activity in inflammatory diseases. Indeed, the possibility to modulate the intracellular metabolic pathways has recently emerged as a potential novel strategy to reshape dysfunctional macrophage functions, offering new therapeutic opportunities (55–58).

For example, several ACLY inhibitors have shown promising results in various therapeutic areas, including the modulation of inflammation and the reduction of atherosclerotic disease progression. (30, 31). Using a model of LPS-induced peritonitis, Lauterbach et al. demonstrated that the treatment with BMS 303141, a specific small-molecule inhibitor of ACLY, determines a decrease in the protein levels of IL-6 and IL-12p70 in both the peritoneum and serum of the BMS-injected mice (30). Moreover, in a recent study from Baardman et al. the role of ACLY in the pathology of atherosclerotic disease was investigated (31). Myeloid ACLY deficiency resulted in stable plaque formation characterized by increased collagen deposition and fibrous cap, as well as a smaller necrotic core. Furthermore, bempedoic acid (ETC-1002), a specific and well tolerated liver-targeting ACLY inhibitor, has been recently approved for clinical use by FDA for the treatment of cardiovascular disease. Similarly, Li et al. showed that the pharmacological inhibition of citrate export (through the mitochondrial citrate carrier (CIC)) inactivated peripheral macrophages and contributes to prevent the formation of cerebral thrombosis (32).

Alternatively, enhancing mitochondrial function, such as through the inhibition of iNOS or through the inhibition of the RNS species generated in inflamed macrophages, could be a valuable strategy to improve the reprogramming of the TCA cycle and effectively control inflammatory diseases. By restoring mitochondrial function, it may be possible to promote metabolic adaptations that can regulate inflammation and restore cellular homeostasis. Van den Bossche et al. demonstrated that inhibition of iNOS in mouse macrophages effectively mitigated the decrease in mitochondrial respiration caused by LPS and IFNg stimuli (14). Consequently, this inhibition led to enhanced metabolic function and facilitated the repolarization of macrophages from a pro-inflammatory phenotype to an anti-inflammatory phenotype. However, the therapeutic application of iNOS inhibitors is limited by their moderate potency and poor selectivity against different isoforms of NOS. Indeed, these inhibitors have shown efficacy in animal models for various diseases, but none have successfully advanced through clinical trials due to concerns about toxicity and selectivity. To overcome these challenges, a deeper understanding of the structural and pharmacophoric requirements for potent and selective iNOS inhibitors is still needed (59). Finally, Seim et al., demonstrated that the pharmacological inhibition of RNS in activated macrophages has a significant impact on restoring the functional lipoic arms and activities of PDH and OGDH, suggesting a potential therapeutical relevance of RNS inhibition for numerous physiological and pathological conditions in which RNS accumulate, such as inflammation, neurodegeneration, and cancer (29).

## Author contributions

MR: Conceptualization, Writing – original draft. FP: Visualization, Writing – review & editing. SG: Conceptualization, Writing – original draft.
